# Supplementary material for: A Likelihood Approach for Real-Time Calibration of Stochastic Compartmental Epidemic Models
Source: PLoS Comput Biol. 2017 Jan 17;13(1):e1005257. doi: 10.1371/journal.pcbi.1005257 (PMC5240920; doi:10.1371/journal.pcbi.1005257)
Supplement: S1 File — (TAR.GZ) [file pcbi.1005257.s014.tar.gz › HSPH_Online-SI-Revision/MSS10/n100-mild/n100-mild_NEW2_table-8.pdf]

1	newly 3 weeks spec	1329.	1459.36	{732., 2362.}	38.3928	0.168018	newly 3 weeks cumul	2670.	2938.59	{1525., 4426.}	36.0461	0.155853	newly 5 weeks spec	2409.	2968.75	{1492., 4288.}	41.9539	0.171344	newly 5 weeks cumul	6876.	8085.26	{4022., 13 294.}	38.6998	0.159037	newly 60 weeks cumul	33 899.	33 849.5	{31 797., 37 109.}	6.80772	0.0348329
2	newly 3 weeks spec	1754.	2949.71	{1833., 4290.}	69.1249	0.209043	newly 3 weeks cumul	4109.	6036.5	{3784., 8703.}	50.3918	0.163775	newly 5 weeks spec	2989.	4719.63	{3477., 5937.}	58.377	0.19081	newly 5 weeks cumul	9407.	14 755.6	{10 200., 20 142.}	57.7274	0.184976	newly 59 weeks cumul	41 142.	34 753.7	{32 424., 36 616.}	15.5276	0.0738862
3	newly 3 weeks spec	1224.	2102.15	{1216., 3017.}	78.3456	0.245468	newly 3 weeks cumul	2588.	4285.47	{2434., 6047.}	72.0406	0.231266	newly 5 weeks spec	2853.	3790.22	{2375., 5353.}	41.3628	0.153877	newly 5 weeks cumul	7485.	11 033.4	{6494., 15 875.}	54.9527	0.190413	newly 62 weeks cumul	35 853.	34 904.8	{32 060., 37 208.}	4.85019	0.0216543
4	newly 3 weeks spec	1047.	21 044.3	{10 458., 35 617.}	1911.36	1.25103	newly 3 weeks cumul	1848.	30 755.1	{13 037., 53 544.}	1565.69	1.1468	newly 5 weeks spec	3916.	14 721.4	{4444., 23 350.}	289.413	0.5748	newly 5 weeks cumul	7888.	68 752.4	{59 104., 81 958.}	772.082	0.928963	newly 66 weeks cumul	49 382.	81 697.1	{67 541., 93 013.}	65.439	0.212444
5	newly 3 weeks spec	1604.	1304.47	{438., 2593.}	44.144	0.258509	newly 3 weeks cumul	3273.	2632.25	{914., 5143.}	41.8408	0.24086	newly 5 weeks spec	2839.	2794.44	{909., 5013.}	40.6467	0.215416	newly 5 weeks cumul	8315.	7426.78	{2749., 14 446.}	39.5711	0.216381	newly 62 weeks cumul	37 686.	32 699.1	{30 694., 36 543.}	13.2962	0.0757997
6	newly 3 weeks spec	1697.	2709.87	{1416., 4184.}	67.6612	0.213235	newly 3 weeks cumul	3762.	5478.16	{3019., 8350.}	54.7469	0.18334	newly 5 weeks spec	3382.	4581.3	{2773., 5870.}	43.1904	0.154253	newly 5 weeks cumul	9658.	13 838.	{7812., 19 797.}	51.1561	0.174926	newly 60 weeks cumul	39 815.	34 294.	{32 091., 36 464.}	13.8667	0.0652874
7	newly 3 weeks spec	621.	2611.85	{712., 7190.}	326.913	0.511386	newly 3 weeks cumul	1186.	3972.96	{1213., 10 131.}	241.42	0.437644	newly 5 weeks spec	2262.	7893.64	{2703., 15 676.}	255.518	0.497655	newly 5 weeks cumul	4678.	17 225.2	{5830., 40 781.}	274.61	0.498276	newly 65 weeks cumul	38 409.	57 832.	{37 096., 70 901.}	52.0373	0.174118
8	newly 3 weeks spec	1914.	2590.53	{961., 3833.}	52.8135	0.207031	newly 3 weeks cumul	4004.	5079.24	{2311., 7701.}	46.2597	0.192463	newly 5 weeks spec	3108.	4735.81	{2890., 6013.}	62.3658	0.225974	newly 5 weeks cumul	9610.	13 584.3	{6591., 18 706.}	54.389	0.203963	newly 59 weeks cumul	33 244.	34 515.	{33 569., 36 705.}	7.66376	0.0368813
9	newly 3 weeks spec	1331.	2100.75	{897., 3096.}	72.3058	0.236586	newly 3 weeks cumul	2488.	3474.27	{1630., 5011.}	55.2737	0.193932	newly 5 weeks spec	3831.	6749.31	{3424., 9929.}	85.7711	0.304932	newly 5 weeks cumul	8579.	14 291.7	{6614., 20 791.}	77.6339	0.255856	newly 64 weeks cumul	48 106.	55 553.6	{36 697., 61 085.}	23.1928	0.113553
10	newly 3 weeks spec	2104.	901.67	{376., 1423.}	57.145	0.455993	newly 3 weeks cumul	4740.	2527.42	{1102., 4032.}	46.6903	0.338807	newly 5 weeks spec	3114.	892.51	{409., 1422.}	71.3388	0.632559	newly 5 weeks cumul	10 319.	4333.94	{2053., 6699.}	58.0004	0.443645	newly 58 weeks cumul	38 689.	10 577.1	{7858., 13 368.}	72.6614	0.60424
11	newly 3 weeks spec	890.	1143.52	{814., 1375.}	36.636	0.147401	newly 3 weeks cumul	1597.	2124.78	{1673., 2501.}	39.9236	0.155703	newly 5 weeks spec	2552.	3280.49	{2019., 3949.}	37.5231	0.152549	newly 5 weeks cumul	5799.	7397.17	{5249., 8787.}	35.9902	0.146174	newly 62 weeks cumul	44 256.	81 173.	{37 670., 92 602.}	87.9628	0.268618
12	newly 3 weeks spec	1981.	2391.26	{1706., 3063.}	26.8773	0.103372	newly 3 weeks cumul	4456.	5182.64	{3723., 6779.}	23.8241	0.0947641	newly 5 weeks spec	3330.	3820.24	{3099., 4418.}	19.809	0.0788481	newly 5 weeks cumul	10 451.	12 180.	{9307., 14 839.}	22.3642	0.0883474	newly 58 weeks cumul	44 615.	33 307.2	{30 720., 36 363.}	25.3454	0.127851
13	newly 3 weeks spec	1116.	1877.05	{667., 2896.}	86.5027	0.26466	newly 3 weeks cumul	2037.	3055.1	{1032., 4634.}	69.8095	0.235789	newly 5 weeks spec	3104.	6104.56	{2211., 9490.}	110.113	0.328246	newly 5 weeks cumul	7098.	12 860.2	{4901., 18 956.}	96.1257	0.294493	newly 63 weeks cumul	40 666.	53 205.	{36 529., 60 666.}	35.1784	0.129169
14	newly 3 weeks spec	1519.	6881.2	{778., 12 427.}	371.227	0.614348	newly 3 weeks cumul	2463.	10 046.5	{1201., 18 210.}	325.738	0.565784	newly 5 weeks spec	4569.	11 108.6	{3621., 15 237.}	156.019	0.421444	newly 5 weeks cumul	9906.	32 335.	{8245., 44 649.}	238.709	0.522976	newly 65 weeks cumul	48 448.	51 762.3	{37 353., 66 292.}	12.2825	0.050239
15	newly 3 weeks spec	1182.	2347.13	{528., 3408.}	128.84	0.308834	newly 3 weeks cumul	2013.	3966.75	{913., 5760.}	123.685	0.304873	newly 5 weeks spec	3065.	4506.92	{1703., 8030.}	70.9305	0.238488	newly 5 weeks cumul	7143.	12 114.	{3731., 24 977.}	95.72	0.276633	newly 62 weeks cumul	48 233.	38 726.4	{35 279., 50 331.}	22.1808	0.109429
16	newly 3 weeks spec	1433.	2957.14	{1788., 4367.}	107.918	0.296869	newly 3 weeks cumul	3454.	6155.23	{3709., 8946.}	80.4809	0.240127	newly 5 weeks spec	2363.	4567.3	{3165., 6062.}	93.6471	0.274157	newly 5 weeks cumul	7772.	14 659.6	{9528., 20 465.}	89.7499	0.26239	newly 58 weeks cumul	32 590.	33 941.5	{31 867., 36 094.}	5.31402	0.0223131
17	newly 3 weeks spec	933.	1205.87	{196., 2099.}	64.582	0.309913	newly 3 weeks cumul	1663.	2203.33	{448., 3731.}	65.801	0.302522	newly 5 weeks spec	2774.	3292.19	{558., 5379.}	57.3926	0.310573	newly 5 weeks cumul	6118.	7595.82	{1555., 12 871.}	60.3645	0.301165	newly 64 weeks cumul	46 517.	38 843.3	{33 949., 54 410.}	29.3413	0.189878
18	newly 3 weeks spec	223.	31 490.8	{10 098., 44 871.}	14 021.4	2.05711	newly 3 weeks cumul	346.	43 735.7	{10 928., 70 857.}	12 540.4	1.98925	newly 5 weeks spec	1979.	9871.07	{1327., 17 677.}	413.542	0.649263	newly 5 weeks cumul	3142.	77 478.4	{60 775., 90 871.}	2365.89	1.37628	newly 68 weeks cumul	44 313.	82 515.9	{67 673., 93 412.}	86.2115	0.264827
19	newly 3 weeks spec	1287.	1073.24	{252., 1941.}	45.6472	0.313818	newly 3 weeks cumul	2744.	2438.95	{678., 3998.}	38.2715	0.242273	newly 5 weeks spec	2837.	1785.98	{365., 3751.}	53.3691	0.452269	newly 5 weeks cumul	7629.	5645.88	{1377., 10 435.}	46.4006	0.326916	newly 59 weeks cumul	38 174.	21 379.9	{8977., 34 219.}	44.0127	0.344084
20	newly 3 weeks spec	216.	2779.96	{4., 4754.}	1253.93	0.940787	newly 3 weeks cumul	340.	3242.66	{17., 5127.}	919.418	0.728024	newly 5 weeks spec	1268.	7423.18	{1., 23 630.}	548.241	1.14454	newly 5 weeks cumul	2188.	16 428.9	{39., 59 063.}	714.362	0.946826	newly 68 weeks cumul	40 762.	67 177.7	{74., 92 951.}	87.8827	0.600998
21	newly 3 weeks spec	570.	14 776.9	{70., 42 192.}	2521.9	1.28408	newly 3 weeks cumul	880.	18 156.6	{120., 51 047.}	1992.21	1.11542	newly 5 weeks spec	2390.	14 809.3	{208., 28 463.}	548.424	1.008	newly 5 weeks cumul	4463.	53 120.1	{506., 89 405.}	1115.91	1.13439	newly 68 weeks cumul	44 593.	72 627.9	{34 813., 93 832.}	79.6598	0.448777
22	newly 3 weeks spec	1853.	2400.77	{1622., 3193.}	36.5219	0.134172	newly 3 weeks cumul	4544.	5247.29	{3561., 6968.}	26.6045	0.105474	newly 5 weeks spec	2912.	3649.92	{2852., 4448.}	30.6889	0.120935	newly 5 weeks cumul	9679.	11 996.	{8782., 15 526.}	30.6386	0.117933	newly 58 weeks cumul	43 141.	31 900.1	{30 416., 34 548.}	26.0561	0.1359
23	newly 3 weeks spec	936.	1372.11	{1132., 1736.}	46.5929	0.160443	newly 3 weeks cumul	1680.	2402.6	{2024., 2954.}	43.0119	0.151031	newly 5 weeks spec	2751.	4543.82	{3761., 6326.}	65.1698	0.210956	newly 5 weeks cumul	5966.	9535.75	{7931., 12 807.}	59.8349	0.197588	newly 63 weeks cumul	45 926.	88 941.7	{87 815., 91 043.}	93.663	0.287002
24	newly 3 weeks spec	678.	22 539.8	{1806., 44 523.}	3226.6	1.31331	newly 3 weeks cumul	1172.	28 379.7	{2150., 58 618.}	2324.23	1.17148	newly 5 weeks spec	2554.	14 850.6	{6403., 27 781.}	485.789	0.708827	newly 5 weeks cumul	4980.	67 341.1	{25 911., 90 427.}	1252.23	1.0826	newly 67 weeks cumul	42 361.	80 698.9	{50 565., 93 997.}	90.5029	0.270824
25	newly 3 weeks spec	1220.	1055.24	{390., 2014.}	43.1164	0.246927	newly 3 weeks cumul	2798.	2608.97	{1051., 4369.}	36.7337	0.196686	newly 5 weeks spec	2397.	1398.99	{449., 3162.}	56.753	0.434697	newly 5 weeks cumul	7013.	5254.92	{2006., 9754.}	43.4436	0.270804	newly 58 weeks cumul	41 718.	16 354.7	{7904., 32 859.}	60.797	0.488704
26	newly 3 weeks spec	1422.	728.32	{280., 1189.}	54.1406	0.399903	newly 3 weeks cumul	2792.	1815.67	{747., 3114.}	42.7074	0.289512	newly 5 weeks spec	3228.	999.74	{295., 1468.}	72.9287	0.651201	newly 5 weeks cumul	8236.	3681.31	{1418., 5713.}	59.8901	0.456175	newly 60 weeks cumul	43 633.	13 659.7	{9563., 15 167.}	68.6941	0.534758
27	newly 3 weeks spec	1429.	2204.33	{1088., 3532.}	65.725	0.205565	newly 3 weeks cumul	3015.	4285.5	{2029., 6785.}	56.1897	0.18701	newly 5 weeks spec	2878.	4279.72	{2305., 6023.}	57.4357	0.192087	newly 5 weeks cumul	7889.	11 848.2	{6087., 17 386.}	59.7357	0.193263	newly 58 weeks cumul	41 619.	35 078.8	{33 086., 37 124.}	15.7144	0.0767574
28	newly 3 weeks spec	1395.	2191.68	{1388., 2967.}	62.1104	0.204388	newly 3 weeks cumul	3462.	4631.18	{2909., 6216.}	42.0745	0.152781	newly 5 weeks spec	2612.	3763.74	{2675., 4777.}	48.6026	0.170026	newly 5 weeks cumul	8023.	11 413.4	{7434., 14 974.}	47.859	0.167948	newly 59 weeks cumul	37 337.	34 735.5	{31 751., 37 060.}	7.21526	0.033194
29	newly 3 weeks spec	1153.	2240.72	{927., 3335.}	106.765	0.313308	newly 3 weeks cumul	2468.	4307.	{1848., 6464.}	87.8177	0.274504	newly 5 weeks spec	2181.	4474.73	{2577., 5805.}	114.14	0.338258	newly 5 weeks cumul	6228.	12 168.8	{5915., 17 011.}	104.776	0.30991	newly 61 weeks cumul	30 741.	34 545.2	{33 717., 36 952.}	17.6667	0.0796337
30	newly 3 weeks spec	1065.	434.25	{44., 845.}	64.7033	0.679549	newly 3 weeks cumul	1901.	864.52	{128., 1695.}	60.2557	0.545714	newly 5 weeks spec	3590.	1038.84	{109., 2147.}	73.3025	0.886189	newly 5 weeks cumul	7402.	2581.67	{343., 5138.}	68.6416	0.69923	newly 63 weeks cumul	47 995.	31 538.	{10 592., 40 494.}	34.5323	0.36409
31	newly 3 weeks spec	1303.	911.47	{226., 1572.}	45.1934	0.309564	newly 3 weeks cumul	2596.	1928.16	{626., 3255.}	40.8837	0.257936	newly 5 weeks spec	2456.	1866.16	{293., 3774.}	53.6637	0.37506	newly 5 weeks cumul	6882.	5142.36	{1160., 8997.}	47.0549	0.309743	newly 60 weeks cumul	37 472.	25 656.4	{9410., 36 434.}	31.6985	0.229626
32	newly 3 weeks spec	2005.	2986.72	{2138., 3787.}	49.9721	0.165913	newly 3 weeks cumul	4624.	6540.01	{4926., 8124.}	42.5011	0.146086	newly 5 weeks spec	3092.	4233.37	{3212., 5174.}	38.206	0.134909	newly 5 weeks cumul	10 135.	14 534.2	{11 092., 18 013.}	44.2749	0.151829	newly 58 weeks cumul	42 820.	32 625.5	{30 477., 34 997.}	23.8078	0.1187
33	newly 3 weeks spec	116.	22 356.2	{1235., 43 704.}	19 175.4	2.07511	newly 3 weeks cumul	183.	29 271.7	{1609., 57 983.}	15 898.	1.95351	newly 5 weeks spec	1068.	14 604.3	{3254., 25 449.}	1276.31	1.08674	newly 5 weeks cumul	1629.	69 038.3	{20 997., 90 412.}	4141.62	1.58509	newly 68 weeks cumul	33 313.	81 452.5	{67 447., 94 000.}	146.506	0.414966
34	newly 3 weeks spec	1093.	1853.76	{349., 3405.}	106.254	0.303218	newly 3 weeks cumul	1962.	3031.02	{660., 5046.}	90.1254	0.278168	newly 5 weeks spec	3687.	4642.3	{1220., 9744.}	64.5066	0.266132	newly 5 weeks cumul	7846.	11 021.3	{2819., 25 662.}	77.6179	0.272969	newly 64 weeks cumul	44 642.	41 716.1	{35 541., 56 225.}	20.5286	0.0905276
35	newly 3 weeks spec	20.	35 910.7	{11 332., 45 050.}	179 456.	3.18162	newly 3 weeks cumul	142.	55 455.2	{12 433., 80 615.}	38 956.4	2.51304	newly 5 weeks spec	625.	5960.33	{522., 11 341.}	865.39	0.865499	newly 5 weeks cumul	970.	80 302.7	{65 266., 91 296.}	8182.38	1.91424	newly 68 weeks cumul	40 997.	83 039.1	{69 670., 93 313.}	104.549	0.342832
36	newly 3 weeks spec	277.	5521.	{0., 21 374.}	1949.09	1.13965	newly 3 weeks cumul	444.	6925.21	{26., 23 036.}	1513.71	0.874592	newly 5 weeks spec	1470.	10 650.6	{0., 27 115.}	682.799	1.26552	newly 5 weeks cumul	2688.	27 179.8	{32., 77 990.}	967.509	1.10092	newly 68 weeks cumul	33 226.	63 509.7	{32., 92 765.}	118.552	0.729199
37	newly 3 weeks spec	1497.	10 911.4	{3460., 15 763.}	628.94	0.80801	newly 3 weeks cumul	2865.	17 583.3	{4788., 27 502.}	515.707	0.72588	newly 5 weeks spec	3432.	9767.02	{5587., 13 563.}	185.289	0.434685	newly 5 weeks cumul	8555.	40 579.4	{29 156., 46 500.}	374.335	0.667455	newly 61 weeks cumul	44 446.	49 191.6	{48 084., 50 430.}	11.072	0.0455979
38	newly 3 weeks spec	250.	27 577.3	{3195., 45 247.}	10 934.6	1.91402	newly 3 weeks cumul	547.	38 399.3	{4395., 73 494.}	6923.67	1.68761	newly 5 weeks spec	1505.	10 257.4	{1069., 17 324.}	595.854	0.817719	newly 5 weeks cumul	2547.	70 949.1	{29 506., 90 797.}	2687.59	1.42399	newly 68 weeks cumul	38 637.	78 029.6	{50 818., 93 368.}	103.955	0.337564
39	newly 3 weeks spec	1338.	10 104.7	{2704., 15 355.}	657.166	0.812408	newly 3 weeks cumul	2487.	15 676.3	{3743., 25 187.}	533.613	0.725713	newly 5 weeks spec	3912.	11 016.7	{6712., 15 162.}	181.922	0.429929	newly 5 weeks cumul	8892.	40 143.3	{28 133., 46 882.}	351.455	0.641595	newly 65 weeks cumul	39 846.	50 655.2	{49 144., 51 511.}	27.1275	0.103582
40	newly 3 weeks spec	586.	11 476.5	{1442., 26 153.}	1862.39	1.17769	newly 3 weeks cumul	886.	15 806.	{1975., 34 951.}	1688.23	1.12131	newly 5 weeks spec	3300.	11 772.7	{6614., 15 617.}	259.059	0.574196	newly 5 weeks cumul	5752.	43 216.7	{21 053., 65 865.}	653.532	0.860332	newly 67 weeks cumul	45 872.	54 844.3	{49 532., 69 009.}	21.9814	0.115615
41	newly 3 weeks spec	397.	13 643.3	{37., 39 813.}	3374.45	1.32501	newly 3 weeks cumul	582.	16 083.3	{68., 46 011.}	2700.09	1.18571	newly 5 weeks spec	2106.	13 210.6	{68., 28 497.}	562.457	0.997044	newly 5 weeks cumul	3564.	48 866.4	{179., 89 063.}	1306.09	1.16141	newly 68 weeks cumul	48 915.	70 339.	{35 388., 93 649.}	64.7051	0.425887
42	newly 3 weeks spec	795.	28 071.5	{19 123., 38 804.}	3433.76	1.53528	newly 3 weeks cumul	1426.	50 787.9	{24 348., 72 454.}	3464.79	1.50239	newly 5 weeks spec	2995.	3075.4	{504., 6162.}	75.7269	0.411342	newly 5 weeks cumul	5937.	65 317.5	{50 862., 79 611.}	1002.26	1.0428	newly 65 weeks cumul	44 109.	67 518.9	{52 298., 80 180.}	55.0717	0.212308
43	newly 3 weeks spec	339.	27 666.1	{5108., 45 024.}	8063.09	1.81105	newly 3 weeks cumul	542.	37 165.7	{5916., 69 056.}	6759.06	1.69151	newly 5 weeks spec	1978.	12 503.4	{1269., 24 102.}	547.139	0.772742	newly 5 weeks cumul	3344.	75 117.6	{55 260., 90 722.}	2148.33	1.34219	newly 68 weeks cumul	47 323.	82 427.9	{68 096., 93 724.}	76.1804	0.27129
44	newly 3 weeks spec	386.	1089.7	{33., 2495.}	247.544	0.647477	newly 3 weeks cumul	600.	1460.73	{78., 3349.}	202.648	0.498172	newly 5 weeks spec	2455.	5063.35	{101., 16 389.}	188.472	0.818513	newly 5 weeks cumul	4090.	9358.54	{223., 28 005.}	205.837	0.668348	newly 67 weeks cumul	47 913.	53 580.6	{14 713., 83 409.}	42.8317	0.358787
45	newly 3 weeks spec	1342.	3313.17	{2051., 4670.}	147.679	0.375244	newly 3 weeks cumul	2662.	5839.54	{3653., 8119.}	120.613	0.327511	newly 5 weeks spec	2863.	7918.19	{4965., 10 114.}	176.746	0.427672	newly 5 weeks cumul	7410.	19 393.4	{13 071., 25 472.}	162.03	0.403226	newly 63 weeks cumul	42 303.	52 135.9	{36 391., 56 543.}	28.5097	0.109298
46	newly 3 weeks spec	594.	9120.14	{520., 23 900.}	1442.64	0.721552	newly 3 weeks cumul	1181.	14 112.5	{970., 39 419.}	1104.64	0.635533	newly 5 weeks spec	2667.	3661.76	{1492., 7448.}	78.0307	0.268309	newly 5 weeks cumul	5207.	24 859.1	{3674., 52 314.}	401.006	0.522019	newly 64 weeks cumul	48 956.	72 181.5	{52 097., 92 348.}	51.0447	0.198976
47	newly 3 weeks spec	763.	5670.56	{1158., 10 472.}	644.668	0.744059	newly 3 weeks cumul	1478.	8031.86	{1821., 15 222.}	445.778	0.617871	newly 5 weeks spec	3193.	13 780.7	{5931., 18 963.}	332.382	0.596391	newly 5 weeks cumul	6436.	32 983.8	{10 879., 45 830.}	413.655	0.655622	newly 64 weeks cumul	48 321.	66 756.	{50 014., 90 698.}	38.1511	0.13092
48	newly 3 weeks spec	1223.	1049.56	{515., 1489.}	30.5691	0.166672	newly 3 weeks cumul	2426.	2149.23	{1096., 2928.}	28.143	0.148643	newly 5 weeks spec	3101.	2248.31	{1069., 3286.}	34.4569	0.218092	newly 5 weeks cumul	7512.	5977.93	{3106., 8407.}	30.5675	0.177219	newly 59 weeks cumul	44 170.	35 490.	{31 793., 40 231.}	20.0719	0.114015
49	newly 3 weeks spec	820.	21 228.7	{14 400., 24 636.}	2490.86	1.41939	newly 3 weeks cumul	1602.	32 630.2	{17 608., 41 951.}	1938.83	1.29345	newly 5 weeks spec	2523.	4304.75	{1593., 8596.}	89.0729	0.265415	newly 5 weeks cumul	5593.	50 576.	{49 241., 52 531.}	806.272	0.982723	newly 65 weeks cumul	35 659.	51 856.8	{51 278., 53 141.}	47.424	0.205724
50	newly 3 weeks spec	648.	9719.01	{3984., 14 044.}	1401.95	1.1113	newly 3 weeks cumul	1146.	14 194.7	{5311., 22 305.}	1141.61	1.02645	newly 5 weeks spec	2869.	11 809.4	{7683., 15 203.}	311.622	0.599824	newly 5 weeks cumul	5616.	40 341.	{32 734., 45 838.}	618.322	0.839831	newly 66 weeks cumul	40 068.	51 117.7	{49 505., 51 498.}	27.5772	0.104896
